# Socioeconomic status and entrepreneurial networking responses to the COVID-19 crisis

**DOI:** 10.1007/s11573-022-01120-w

**Published:** 2022-11-22

**Authors:** Leif Brändle, Helen Signer, Andreas Kuckertz

**Affiliations:** Entrepreneurship Research Group, University of Hohenheim, Wollgrasweg 49, 70599 Stuttgart, Germany

**Keywords:** Networking, Entrepreneurship, COVID-19, Socioeconomic status, Social class, M13, D85, D91, L26

## Abstract

Networks play a vital role for entrepreneurs in overcoming crises. The most vulnerable to crises are those from lower socioeconomic backgrounds. However, we know less about the role of socioeconomic status in entrepreneurial networking. This study investigates whom entrepreneurs call in case of emergency. We develop hypotheses on how entrepreneurs’ socioeconomic status influences models of networking agency in situations of economic threat. The results of a pre-registered randomized experiment in the COVID-19 context conducted with 122 entrepreneurs from the US indicate that entrepreneurs in higher socioeconomic status positions activate contacts to serve their own goals (i.e., independent networking agency) when facing an economic threat. In contrast, and counter-intuitively, entrepreneurs of lower socioeconomic status are more likely to support others when facing an economic threat (i.e., interdependent networking agency). Exploring the evolving network structure, our explorative post-hoc analyses suggest that entrepreneurs activate closer networks (i.e., higher density and stronger ties) under threat. The study discusses the implications of these findings for the theory of entrepreneurial networking in general and network responses to crises in particular.

## Introduction

“I found a way to thrive during the pandemic, being very active in communication with many people […]”

“I lost all of my savings and nearly became homeless. […] Friendships dissolved due to distance. Very isolated.”^1^

Entrepreneurial networks are possibly the most critical source of support in economic crises (Pollack et al. 2012; Doern et al. 2019; Muñoz et al. 2019). But whom do entrepreneurs call in case of emergency? The global COVID-19 pandemic has posed a severe threat to entrepreneurs and their ventures around the globe (Brown and Rocha 2020; Kuckertz et al. 2020; Zahra 2021). Without a functioning social support system, entrepreneurs struggle fiercely to navigate through such an economic crisis (Giones et al. 2020; Ratten 2020). There is implicit consensus among scholars of different theoretical camps on the importance of entrepreneurial networks for opportunity recognition and the mobilization of resources (Sorenson and Stuart 2001; Hoang and Antoncic 2003; Bhagavatula et al. 2010; Semrau and Werner 2014). A traditionally dominant literature stream on entrepreneurial networks has focused on a structuralist perspective in which initial positions determine future network outcomes (e.g., Stam and Elfring 2008; Milanov and Shepherd 2013). In contrast, a growing stream of entrepreneurship research emphasizes an agentic perspective of entrepreneurial networks (Hallen et al. 2020), paralleling developments in sociological network theories (Emirbayer and Goodwin 1994). Studies in this theoretical tradition portray entrepreneurs as the agentic architects of their networks based on their intentional decisions to form new and interact with established ties (Hallen and Eisenhardt 2012; Vissa 2012).

More recently, a third theoretical camp has emerged, which proposes that the unique context of entrepreneurial uncertainty (McMullen and Shepherd 2006) leads to idiosyncratic modes of entrepreneurial networking (Engel et al. 2017; Kerr and Coviello 2020). This research concludes that, since outcomes in entrepreneurial environments are hard to predict, specific networking outcomes are not at the center of social interactions in entrepreneurial networking. Instead, entrepreneurs mainly base their social interactions on the joint generation of serendipitous objectives and reciprocal value creation (Engel et al. 2017; Ahoba-Sam and Charles 2019; Kerr and Coviello 2020; Busch and Barkema 2022a, b). As such, entrepreneurial networking contrasts models of agency involving *goal-directed networking* (social ties as an instrument to a predefined objective: Hallen and Eisenhardt 2012; Vissa 2012) versus *effectual networking* (social ties to generate serendipitous objectives based on reciprocity: Sarasvathy 2001; Engel et al. 2017) and ultimately starts a conversation about what constitutes networking agency in entrepreneurship.

While increasing our awareness of how uncertain environments alter entrepreneurial networking (e.g., goal-directed vs. effectual) represents a major advancement to previous discussions on agency and structure in entrepreneurial networking, less attention has been given to the role of entrepreneurs’ social self and its influence on agency in entrepreneurial networking. This is important for several reasons. First, entrepreneurial networking implicitly involves interactions between humans. While these individuals make decisions on whom to contact based on a myriad of objective and subjective factors (such as their position in the network, the situation, and their goals), one decisive yet often forgotten explanation for their actions in an entrepreneurial network is their personal and socialized understanding of agency in social interactions. To this end, prior research has documented that an individual’s position in a social hierarchy leads to fundamentally different modes of interactions with others (Snibbe and Markus 2005; Gruenfeld et al. 2008; Kraus et al. 2009). Second, individuals make sense of specific situations differently, based on who they are (Weick 1995). Since networking occurs through individual decisions of activating and mobilizing specific contacts based on perception and judgment, it is socially constructed (Mannucci and Perry-Smith 2021; Nai et al. 2021). Hence, individuals’ understanding of their self within a social structure is crucial for understanding their social construction of a networking response. Third, their decision on how to deal with a specific environmental cue depends on the personal situation individuals find themselves in. Specifically, situations of economic threat are unequally distributed across society. Those at the lower ends of a social hierarchy perceive additional strain due to economic crises such as the one followed by COVID-19 (Munir 2021; Bapuji et al. 2020b). In sum, considering the interaction between environmental and personal strains might shed further light on individual networking responses (Smith et al. 2012).

The purpose of this study is to investigate whom entrepreneurs contact in situations of threat. Specifically, reflecting the economic consequences of the COVID-19 pandemic, the study investigates how the subjective socioeconomic status of entrepreneurs (i.e., their subjective rank in a societal hierarchy) influences their goal-directedness in networking under threat*.* Our main argument is that entrepreneurial networking depends on the interaction between environmental situations and individual interpretations based on entrepreneurs’ subjective socioeconomic positions. Specifically, this study draws on theories of agency in social interactions and socioeconomic status (Kraus et al. 2009) in order to explain and show how goal-directedness in entrepreneurial networking (Engel et al. 2017) changes based on the combination of situational threat and a network actor’s social position. The study argues that entrepreneurs of higher socioeconomic status exert goal-directed networking behavior during situations of threat due to their relative independence from individual network contacts. Entrepreneurs of lower socioeconomic status are more likely to depend on their network's reciprocity and are thus more likely to activate contacts to support them in situations of threat.

While the study employs a randomized preregistered experiment including a control and treatment group to test its hypotheses, we still emphasize the exploratory nature of the research. We test our hypotheses in an online experiment with 122 entrepreneurs on the prolific platform. Despite the advantages of the platform compared to other panel providers (Eyal et al. 2021), there is a need for further experiments and correlational studies to test the external validity of this study’s results. Furthermore, particularly in our post-hoc analyses, we encounter unexpected results subject to further investigation. Considering the limitations and strengths of our experiment, the study makes several theoretical and methodological contributions to extant literature. First, the study contributes to research on entrepreneurial networking by proposing the socioeconomic status of entrepreneurs as a boundary condition to entrepreneurial networking behavior. Second, the study introduces a measurement for and testing explanations of why and when entrepreneurs apply more goal-directed networking tactics. Third, the study contributes to research on the response of entrepreneurs to adversarial situations by emphasizing how external situations influence decisions on which sub-networks within an entrepreneurial network to activate. Understanding whom entrepreneurs contact in a crisis and why is essential as it might shed light on how and when entrepreneurs adapt their entrepreneurial networking. That aspect of the research informs the general crisis literature on entrepreneurship and, more specifically, the emerging literature on the socioeconomic consequences of the COVID-19 pandemic.

## Theoretical background

### Entrepreneurial networking

Entrepreneurial networking is “what entrepreneurs do in creating and shaping network ties” (Engel et al. 2017, p. 37). Entrepreneurial networks can include a wide variety of relationships such as family and friends, venture capitalists (Sapienza 1992; Sorenson and Stuart 2001), customers, competitors, or suppliers (Street and Cameron 2007). Prior literature has identified several benefits for the engagement of entrepreneurial firms in entrepreneurial networks. Social interaction in entrepreneurial networks mainly fosters the identification of entrepreneurial opportunities and access to relevant resources (Sorenson and Stuart 2001; Hoang and Antoncic 2003; Semrau and Werner 2014). The access to knowledge and information in entrepreneurial networks strongly influences innovation outcomes. Opportunities are developed in and diffuse among the interaction between network actors.

The network positions of entrepreneurs influence the benefits they can derive from their social relationships (Freeman 1978; Burt 1982, 2004; Stam and Elfring 2008; Ebbers 2014) and their future role in these networks (Milanov and Shepherd 2013). For instance, an entrepreneur connected with more (less) diverse networks, in which network members are sparsely (densely) connected, typically creates more (less) novel opportunities (Burt 2004) and enhances (hampers) firm performance (Stam et al. 2014). Nevertheless, this structural perspective implies that the entrepreneur’s role in the entrepreneurial network is static (see Hallen and Eisenhardt 2012; Vissa 2012). However, entrepreneurial agency involves the transformation of existing structures (Stevenson et al. 2020; McMullen et al. 2021).

To this end, a growing research stream focuses on the networking agency of entrepreneurs (e.g., Vissa 2011, 2012; Hutter 2014; Engel et al. 2017; Hallen et al. 2020; Tasselli et al. 2020). That networking agency behavior is generally understood as the purposive action of actors serving the assertion of their interests (Emirbayer and Goodwin 1994). Researchers agree that an individual’s position within a network both constrains and promotes the actions of that actor (e.g., Stevenson and Greenberg 2000; Tasselli et al. 2020), but “people [not networks] are the source of action “(Burt 2012, p. 545). Entrepreneurs create their position in the network by interacting with other actors and thus building, maintaining, or changing relationships (Slotte-Kock and Coviello 2010). A duality emerges, whereby the network simultaneously becomes structuring while being structured by the actors (Tasselli et al. 2020).

A fundamental assumption of networking agency is that the selection and evaluation of potential network partners are *goal-directed*. In other words, networking is based on the extent to which partners possess resources that are important to their personal goal fulfillment (Fitzsimons and Shah 2008; Gruenfeld et al. 2008; Vissa 2011; Hallen and Eisenhardt 2012; Hutter 2014; Shea and Fitzsimons 2016; Hallen et al. 2020). Hallen and Eisenhardt (2012) refer to entrepreneurs connecting with a desirable partner with little effort and time to act responsibly with limited resources as efficient tie formation. New ties are formed by matching the entrepreneur’s current projects and the opportunities and resources offered by the contact with the new person (Vissa 2012). In this agentic view, entrepreneurs are portrayed as “heroic architects who strategically search, plan, and pursue their predefined goals” (Engel et al. 2017, p. 36). According to these ends, entrepreneurs strategically select ties and rearrange their networks based on their personal goals.

In contrast, an emerging view on entrepreneurial networking suggests that, since outcomes in uncertain environments may be unknown to the entrepreneur (Sarasvathy 2001), the interaction with an existing contact is focused on the joint creation of value and not on instrumental motives, especially not directly on the added value that contact can offer (Engel et al. 2017). Therefore, individuals self-select themselves in the network based on their commitment and are not selected based on potential future value (Engel et al. 2017; Galkina and Atkova 2020; Kerr and Coviello 2020). Unexpected contingencies can arise by discovering new facets in existing ties and through contact with new people (Busch and Barkema 2022a, b). This *effectual networking* can lead to a changed network but also to the appearance of serendipitous new goals (Engel et al. 2017; Ahoba-Sam and Charles 2019; Kerr and Coviello 2020). Through these interactions with the environment, entrepreneurial outcomes emerge and change (Sarasvathy 2001; Alvarez and Barney 2007; Welter et al. 2016; Engel et al. 2017; Kerr and Coviello 2020). As a flexible goal is assumed, it contrasts with goal-directed networking, where networking preferences, the goal, or the target are known.

One of the main contributions of this stream of research is integrating the insight of structural influences into a model of agentic networking. The key argument is that characteristics of entrepreneurial environments shape how entrepreneurs form and shape ties. Since the demands of entrepreneurs shift quickly to parallel the growth of their ventures and the exceptional dynamic of the environments in which they are embedded, the answer to who is the right contact to call is in flux (Witt 2004; Slotte-Kock and Coviello 2010). In other words, entrepreneurs only benefit from their networks if they can adapt network configurations to new circumstances (Maurer and Ebers 2006). Exploiting the advantages of a network position requires actors to recall helpful contacts in specific situations (Smith et al. 2012; Shea and Fitzsimons 2016). Nevertheless, prior research put less emphasis on how exogenous situations change an entrepreneur’s cognitive network activation. For instance, Kerr and Coviello (2020) note that effectual networking ignores the structure surrounding the entrepreneur. Hence, we elaborate on the role of economic crises for entrepreneurial networks in the following section.

### Networking responses to situations of threat

One of the most impactful environmental situations for social interactions is crises. A crisis is a temporal situation that implies significant negative consequences for those actors involved. One of the consequences of crises is threats that can take various forms. For instance, while the COVID-19 pandemic as a crisis represents a threat to individual health, economic threats follow from resulting crises in businesses. Prior literature has discussed the impact of several crises on entrepreneurs and their responses, most notably the Wall Street Crash and the subsequent great depression of the early 2000s, Brexit, and, more recently, the multifaceted crisis triggered by COVID-19 (Brown and Rocha 2020; Tsilika et al. 2020). Because entrepreneurs have a key role as innovators in an economy (Schumpeter 1934), they play a twofold role in such severe crises. First, they are an integral part of the solution by fostering innovation to aid in rebuilding after a crisis (Doern et al. 2019; Ebersberger and Kuckertz 2021) and stimulate economic growth (Brown and Rocha 2020; Cannavale et al. 2020). Entrepreneurs also, however, face severe threats to their commercial survival as a result of an economic crisis like that following the COVID-19 pandemic (Kuckertz et al. 2020). In order to adequately respond to economic threats, entrepreneurs must develop resilience in times of crisis (Doern et al. 2019; Williams et al. 2017). To this end, the rearrangement of existing resources and the mobilization of additional ones becomes crucial to firm survival during a crisis (Wenzel et al. 2021).

When entrepreneurs face severe resource constraints, support systems are critical to their crisis response (Doern et al. 2019; Lefebvre 2020; Morris 2020). While their established rivals struggle to adjust to exogenous shocks, entrepreneurs find ways to adapt to and even benefit from crisis-induced uncertainty (Davidsson 2021; Kuckertz and Brändle 2021). Entrepreneurs engage with their stakeholders to mobilize resources that generate new opportunities from uncertainty (Sarasvathy 2001, 2008). Effectuation theories of entrepreneurship recognize that social interactions are central to their outcome (Sarasvathy 2001; Fisher 2012; Engel et al. 2017). Acquiring support from others helps entrepreneurs overcome resource-constraint environments and provides a means to identify new entrepreneurial opportunities.

Multiple studies mention the crucial role of social support from others during the COVID-19 pandemic, support that bolsters the resilience of entrepreneurs and boosts the chances of their ventures surviving (Giones et al. 2020; Kuckertz et al. 2020; Ratten 2020). For instance, Giones et al. (2020) find that emotional support has played a crucial role in the perseverance of entrepreneurs during the COVID-19 crisis. Kuckertz et al. (2020, p. 3) investigate the crisis response by entrepreneurs concerning COVID-19 and report that entrepreneurs relied on relational capabilities and “combining available internal resources and calling upon external resources from their network (Baker and Nelson 2005), which would include the goodwill of partners, mutual support in the startup community, and access to social capital through brokers.” Nevertheless, while these studies point to the vital role of social support, research on entrepreneurial networking in crisis is scarce. Furthermore, since networking agency involves purposive decisions of individuals, their sensemaking of the situation likely informs networking responses.

A consistent finding across crises is that they reproduce social inequality. The consequences of economic crises are more likely to influence individuals in less privileged positions (Pfeffer et al. 2013; Kantamneni 2020). During COVID-19, individuals in SN lower socioeconomic status positions have been faced with relatively more severe threats to their health and economic survival (Munir 2021; Bapuji et al. 2020b). Emerging evidence indicates that entrepreneurs in less privileged social positions struggle most to cope with the consequences of COVID-19 (Kuckertz and Brändle 2021). Hence, we explain in the following section why socioeconomic status is a critical explanation of how individuals interact with others in threatening situations.

### The role of socioeconomic status in networking agency

Economic inequalities pose serious threats to societies and organizations around the world (Côté 2011; Amis et al. 2020, 2021; Pitesa and Pillutla 2019; Kish-Gephart et al. 2022). Given societal dispersion in income and wealth, individuals face unequal access to resources and opportunities (Bapuji et al. 2020a; Chancel et al. 2022). As such, the objective access to material wealth and their subjective perceptions of socioeconomic status form the social class individuals are born into or acquire over time (Adler et al. 2000; Kraus et al. 2009; Côté 2011).^2^ Individuals from lower social class backgrounds earn less compared to their colleagues in the same position (Laurison and Friedman 2016) and face additional barriers to climbing the career ladder (Pitesa and Pillutla 2019; Ingram and Oh 2022). At the same time, individuals transitioning from working-class backgrounds bring unique strengths to organizations such as bridging cultural differences and being empathic leaders (Martin et al. 2016; Martin and Côté 2019).

The objective resource endowment and consequential subjective perception of rank vis-à-vis others in society shape how individuals think, feel, and act (Bourdieu 1984; Loignon and Woehr 2018) which has significant consequences on individual modes of agency (e.g., Snibbe and Markus 2005; Kraus et al. 2009, 2012; Gustafsson 2012; Kraus and Stephens 2012; Stephens et al. 2014). As individuals in lower socioeconomic positions find themselves in situations of resource scarcity, they learn to be dependent on the external environment in order to better cope with constraints (Kraus et al. 2009). Accordingly, disadvantaged positions increases individuals’ orientation toward the community (Rucker et al. 2018). Instead, individuals in higher socioeconomic positions internalize the experience to be able to achieve goals independently due to their access to relevant resources. For instance, individuals’ socioeconomic status positively influences the focus on their self regarding the mastery of goals (Kraus et al. 2009, 2012).

While there is an increasingly solid ground on socioeconomic status differences in social relationships that highlights the other (i.e., interdependence) vs. self-orientation (i.e., independence) in social interactions (Piff and Robinson 2017), only a few studies investigate networking differences (e.g., Smith et al. 2012) and, to the best of our knowledge, there is no study that has considered socioeconomic status in entrepreneurial networking. In the following hypotheses, this study argues, based on the concepts above, that the subjective socioeconomic status of entrepreneurs shapes their networking response to situations of economic threat.

## Hypotheses

### Entrepreneurs’ goal-directed networking behavior under threat

Against the reviewed literature in the study’s theoretical background, situations of threat might alter entrepreneurial networking. Specifically, we expect entrepreneurs to more likely activate contacts based on their usefulness (i.e., instrumentality) to fulfill specific goals for the following reasons.

While a crisis might heighten uncertainty about the future (Bergenholtz et al. 2021), the first responses to imminent threats may be less ambiguous. Missing clarity on the need to satisfy specific goals is a prerequisite for effectual networking (Engel et al. 2017). Engel et al. (2017, p. 41) state that “as long as ambiguity about what to do next is dominating entrepreneurial decisions, our model holds” (p. 41). In turn, this means that when responses can be defined more clearly, existing goal-directed models of networking apply. That is, entrepreneurial actors form and shape ties based on the contacts’ propensity to serve a predefined function that benefits the entrepreneur or the venture. This seems particularly plausible in times of economic threat when the imminent financial pressure to survive gains importance relative to long-term developments (Wang et al. 2021). As several studies indicate, entrepreneurs and their ventures search for financial backing and emotional support when facing a threat (Giones et al. 2020; Kuckertz and Brändle 2021). Hence, the potentially beneficial function of existing network ties within the phase of economic threat might be more explicit, which allows for a more instrumental approach to networking.

Second, ventures currently or perspectival affected by an economic threat focus on purposefully using their constrained resources which explicitly includes more efficient entrepreneurial networking (Lefebvre 2020; Morris 2020). As in an effectual networking approach, networking outcomes are based on contingencies; they always involve the risk of sunk costs. That is, forming and shaping ties with contacts in the network and making pre-commitments (Engel et al. 2017) do not guarantee any beneficial outcomes for a network actor. We argue that entrepreneurs are more likely to aim to decrease network actions that are not linked to specific outcomes under economic threat. For instance, several studies on crises report that entrepreneurs and their ventures focus on their core business when facing economic threats (Kuckertz et al. 2020). This would exclude any networking activities that are primarily based on serendipitous outcomes. In contrast, under these circumstances, networking would aim to support entrepreneurs’ short-term and predefined value creation activities (Shea and Fitzsimons 2016).

Taken together, we argue that under economic threat, entrepreneurs switch to more specific goals (e.g., to ensure solvency) and are more likely to weigh their resource commitments in social interactions against the background of efficiency. In consequence, entrepreneurs might be more likely to approach their contacts based on how these contacts contribute to the fulfillment of specific goals.

*H1: In situations of economic threat, entrepreneurs implement more goal-directed networking.*

### Socioeconomic status and entrepreneurial networking under threat

The study’s main argument is that entrepreneurs’ socioeconomic status influences their networking responses to a situation of economic threat. We posit that higher socioeconomic status leads to increased goal-directed networking under threat, whereas lower status decreases goal-directed networking. In the following, we unfold several reasons that lead to our hypothesis.

While agency for individuals from lower social status positions resides within their community, higher status individuals perceive agency in their independence. For instance, students from lower socioeconomic backgrounds perform better in teams (Dittmann et al. 2020). Higher-status individuals are more likely to extract benefits from the network (Menon and Smith 2014; Smith et al. 2020) and are more assertive in tie-formation (Smith et al. 2012). Accordingly, those in higher social positions are more confident in forming ties that will generate personal gains, whereas those in lower social positions instead form communal orientations and increasingly empathize with the needs of others (Kraus et al. 2012; Townsend and Truong 2017).

Situations of threat strengthen the differences in the mode of agency between individuals in higher vs. lower socioeconomic status positions (Galinsky et al. 2003; Stephens et al. 2009; Griskevicius et al. 2011; Smith et al. 2012; Mittal and Griskevicius 2014). For instance, individuals from lower socioeconomic status backgrounds activate smaller and more constrained networks when facing a job loss threat (Smith et al. 2012). Stephens et al. (2009) studied business owners categorized as leavers or stayers in the aftermath of Hurricane Katrina in New Orleans in 2005. While the leavers were predominantly middle status, with higher incomes, more extensive social networks, and better education, the stayers were mostly low status with contrasting characteristics. Nevertheless, stayers described themselves as interdependent, caring for others, and strong together (Stephens et al. 2009).

Finally, higher socioeconomic status is closely tied to power which enables one-sided contributions in relationships (Anderson and Galinsky 2006; Stephens et al. 2009). To these ends, social positions translate into the opportunities individuals draw from the network (Smith et al. 2020). In several experiments, Anderson and Galinsky (2006) show that individuals primed with superior positions act more goal-oriented. In other words, they view other individuals more instrumentally based on how useful they are to them (Gruenfeld et al. 2008). To this end, entrepreneurs of higher socioeconomic status might be able to substitute contacts that do not fulfill a beneficial function, while entrepreneurs of lower social status are more likely to depend on the reciprocity of their network (Kraus et al. 2009). They are thus more likely to avoid social risks and base their networking on reciprocity (Anderson and Galinsky 2006). The supportive caring of network contacts during threats builds the foundations for service in return.

In sum, the above arguments suggest that entrepreneurs of higher socioeconomic status use their privileged positions in the face of an existential threat to extract benefits from their relationships. This is in line with a model of networking agency that is based on independence. However, entrepreneurs from lower socioeconomic situations lack the position to enforce the support of their network ties. Instead, they will, in line with network agency based on interdependence, increasingly focus on the needs of others, temporally defer their personal interests, and count on future reciprocity when they need it.

*H2: The higher the socioeconomic status of an entrepreneur, the more goal-directed the networking under threat conditions.*

## Method

To test the hypotheses mentioned above, we conducted a randomized experiment. We pre-registered our hypotheses with *As Predicted* and had our research design and data collection approved by our university’s ethics committee. Experiments are increasingly used in entrepreneurship research (for a review, see Stevenson et al. 2020), social class research (e.g., (Jetten et al. 2017), and network research (e.g., Mannucci and Perry-Smith 2021). Experiments are beneficial for discovering and measuring causal relationships, especially when they are difficult to isolate using other methods. One of the potential strengths of an experiment is the random assignment of participants to different conditions, either through natural occurrences or by actively influencing participants (Stevenson et al. 2020). Random assignment eliminates the effect of unobserved variables, including biases or environmental conditions, on the dependent variable and isolates the effect of the variable under consideration (Hsu et al. 2017).

### Sample and procedure

The randomized experiment manipulates the economic threat arising from the COVID-19 crisis and measures its causal consequences for goal-directed network activation among entrepreneurs. Following Hsu et al. (2019), we collected data at two points in time in April and May 2021 (see Tables 1 and 2). In the first session (t_0_), we collected the demographic characteristics of the participants and set up several control variables, such as the years worked in their company and the number of employees reporting to them. The basic values of the variables under consideration were measured in t_0_, which are the baseline attitudes toward entrepreneurship and their current network (Hsu et al. 2019). The process makes it possible to assess whether participants' random assignment and manipulation were successful. While this first part in t_0_ was the same for all participants, in t_1,_ they were randomly divided into two groups, the *threat* condition and *no threat* (or control) condition, respectively. To avoid any spillover effects, t1 took place one week after t_0_ (Hsu et al. 2019). At the end of t_1_, participants had to guess the topic of the survey to avoid knowledge about the topic influencing the survey results (Stevenson et al. 2020). After completing the questionnaire, participants were informed that the manipulation was fictional to restore their original psychological state and thanked for their participation (Hsu et al. 2017).

**Table 1:** Overview of the experimental procedure

Session 1: Pre-experimental online survey in t_0_	
Demographics (age, gender, socioeconomic status by Adler et al. (2000)) Control variables (number of employees, years in business, work satisfaction, industry)	
Threatened_0_: “Please rate to which degree do you feel threatened in your work life at this moment.” (1 = not at all threatened, 7 = very threatened)	
Network_0_: “From time to time, people informally discuss any range of matters. Thinking about your own network. How many people would that be?” (in numbers)	
Networking_0_: CapabilityNetworking_0_, FrequencyNetworking_0_, UsefulnessNetworking_0_	
Session 2: online experiment in t_1_ (one week after Session 1) Participants were randomly assigned to one of two experimental groups (high and no threat group)	
Variable measured:	
Socioeconomic status (Adler et al. 2000)	
High threat condition	No threat condition
Fictitious article describing the effect of the COVID-19 pandemic on small businesses-19	Fictious article describing the end of the COVID-19 pandemic and economic recovery
“Imagine that you founded a business which was doing well before the crisis. Suppose that one year later, because of the COVID-19 pandemic, you will lose your customers and revenue. As a consequence, there is a risk your business might go bankrupt	“Imagine that you founded a business which was doing well before the crisis. Even though the COVID-19 pandemic hit the economic world, your business did not lose customers or revenue. Now that COVID-19 is over, you no longer run the risks caused by the pandemic
Imagine for a moment what this might feel like and write a few sentences below. *(2–3 sentences)”*	Imagine for a moment what this might feel like and write a few sentences below. (2–3 sentences) “
After the manipulation, two manipulation check questions were asked, just before measuring the activated network and networking:	
Measure the degree they feel threatened (Turner et al. 1992)	
“Were you told in the news article that the COVID-19 crisis is over?” Response categories: yes/no	
Dependent Variables measured: Activated network_1_: “Who are the people you will contact to support you with such business-related matters you just described?	
Name each person individually. Just use initials. *(list as many as you want, maximum of 10)”*	
In addition, it was asked whether they would contact a person for emotional support, advice/career/professional support or financial support and if the contacts are friends and family or business contacts	
Next, participants should describe the strength of their own relation to the contacts named and further between the network contacts themselves	
Goal-directed networking_1_: seven items adapted by Gruenfeld et al. (objectification scale, 2008)	
Restorage of psychological state and remuneration

**Table 2:** Constructs and items of the experiment

	Constructs t1	Scale	References
1	Socioeconomic status*—*Please think about your social class At the top of the ladder are the people who are the best off, those who have the most money, most education, and best jobs. At the bottom are the people who are the worst off, those who have the least money, least education, worst jobs, or no job. Please place an ‘X’ on the rung that best represents where you think you stand on the ladder.)		(Adler et al. 2000)
2	Threatened*—(Thinking about the article and yourself owning a small business, please rate to which degree you feel…)*	*Seven-point scale*	(Turner et al. 1992)
2.a	.. Comfortable (1) or uncomfortable (7)		
2.b	.. Calm (1) or shaky (1)		
2.c	… Secure (1) or tense (7)		
2.d	… Confident (1) or panicky (7)		
2.e	… Relaxed (1) or frightened (7)		
3	Goal-directed networking*—(Based on the situation described in the article, please state your agreement with the following statements.)*	*1* = *strongly disagree to 4* = *neither agree nor disagree to 7* = *strongly agree*	(Gruenfeld et al. 2008)
3.a	I think more about what my contacts can do for me than what I can do for them		
3.b	I tend to contact people only when I need something from them		
3.c	I try to motivate my social contacts to do things that will help me succeed		
3.d	Networking is important to me because it helps me accomplish my goals		
3.e	I get in touch with people based on how useful they are to me		
3.f	My relationships with people are based on how much I enjoy our relationship, rather than how productive our relationship is (R)		
3.g	If the nature of my work changed and one of my contacts wasn’t helpful to me anymore, the relationship probably wouldn’t continue		

To enhance the external validity of the study results, the sample needed to represent entrepreneurs and their network response to the COVID-19 pandemic (Hsu et al. 2017; Stevenson et al. 2020). Achieving a suitable sample of entrepreneurs for the experimental or control group in entrepreneurship research is challenging (Markman et al. 2005). We invited entrepreneurs to participate in the experiment via the online research platform *Prolific*. Entrepreneurs received financial compensation for their participation. *Prolific* has been used frequently in rigorous past behavioral research (Gérain and Zech 2018; Gunia et al. 2021; Marreiros et al. 2017; Sherf and Morrison 2019), including social class research (Callan et al. 2017) and research on the consequences of the COVID-19 pandemic (Wise et al. 2020). The research platform is characterized by a low dropout rate, a high response rate, and high data quality (Eyal et al. 2021). Compared to other platforms, participants are characterized by a higher income level and lower dishonesty (Peer et al. 2017). The master data of *Prolific* allows precise prescreening (Palan and Schitter 2018). Hence, we ensured that only currently active business owners participated in the experiment. Furthermore, to avoid national differences (e.g., in the impact of COVID-19 measures, networking, or the role of social inequality), we focused on entrepreneurs from the United States since the USA constantly ranks highly in terms of entrepreneurial activity (GEM 2019/2020) and social inequality (Wodtke 2016).

A total of 189 entrepreneurs contributed at both time points of the experiment (t_0_ and t_1_). Since 240 participants contributed at t_0_, this corresponds to a response rate of 78.75%. Assuming that a certain processing time is necessary to answer the questions meaningfully, all submissions that fell below a minimum response time were deleted. Those failing the manipulation check were removed as well. Therefore, the final sample comprised 122 participants, all of whom described themselves as entrepreneurs, either founders or co-founders of a company. Of these final participants, 53 (43.4%) were female, and 66 (54.1%) were male, and three participants (2.5%) did not assign themselves to either gender. The average age was 40.87 years, ranging from 19 to 78 years. On average, the respondents had an average of 8.5 employees. A question on socioeconomic status hierarchy (Adler et al. 2000), led to 70 participants (58.6%) assigning themselves to the upper half and 52 (42.6%) to the lower half of the scale. A college degree (57.4%) was the most common educational attainment, and most respondents declared an income greater than USD 50,001 (66.4%). Overall, participants were satisfied with their jobs (M = 4.80, SD = 1.466) and did not feel threatened (M = 2.45, SD = 1.667). The informants were confident of their networking (M = 4.79, SD = 1.764) and were often involved in it (Mf = 4.48, SD = 1.759). Hence, they found that networking was a useful element of their business success (M = 5.35, SD = 1.744).

### Manipulation of economic threat due to COVID-19 a crisis

The threat manipulation was conducted using two fictional online articles, followed by an open-ended question on how the participants felt about the economic threat, including their company's potential loss. Both the manipulation of emotions (Smith et al. 2012, p. 75) and the uncertainty manipulation using news articles (Griskevicius et al. 2011; Mittal and Griskevicius 2014) are established methods. Similar imagination tasks have been utilized to manipulate threats during the COVID-19 pandemic (Wang et al. 2021). We pre-tested the experimental procedure and manipulation before the actual experiment and adjusted the manipulation based on the feedback from the participants.^3^

**Fig. 1:**
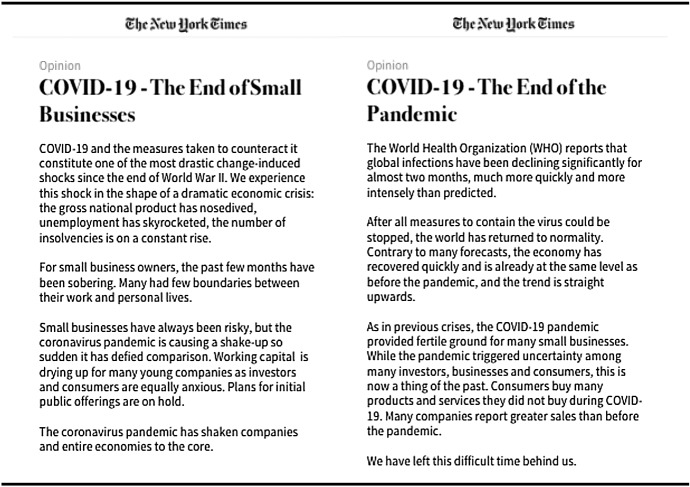
Overview of the threat manipulation by two fictitious newspaper articles

The fictional newspaper articles were presented to manipulate the perceived threat level (see Fig. 1). Participants were informed that the article had recently appeared in the New York Times. Following Mittal and Griskevicius (2014), both texts were precisely the same length and formatted to look like a New York Times online article. While the article of the manipulation group described the strong impact of the COVID-19 pandemic on small businesses to trigger the emotion of feeling threatened, the control article described the end of the COVID-19 pandemic. The following overview reproduces the newspaper articles used to manipulate the subjective threat level. The text for the threat manipulation is on the left (*threat* condition), while that for the control group (*no threat* condition) is on the right.

Having read their respective threat conditions article, the participants were tasked with writing two or three sentences expressing their feelings (Smith et al. 2012, p. 75):

Condition high threat: *“Imagine that you founded a business which was doing well before the crisis. Suppose that one year later, because of the COVID-19 pandemic, you will lose your customers and revenue. As a consequence, there is a risk your business might go bankrupt. Imagine for a moment what this might feel like and write a few sentences below.”*

Condition no threat: *“Imagine that you founded a business which was doing well before the crisis. Even though the COVID-19 pandemic hit the economic world, your business did not lose customers or revenue. Now that COVID-19 is over, you no longer run the risks caused by the pandemic. Imagine for a moment what this might feel like and write a few sentences below.”*

Following the fictional text regarding the COVID-19 situation and the subsequent description of feelings, participants rated how threatened they felt using Turner et al.’s (1992) 5-item scale. This process evaluated if the manipulation altered participants’ feelings as anticipated. These items were measured on a 7-point scale that featured the following poles: comfortable (1) versus uncomfortable (7), calm (1) versus shaky (7), secure (1) versus tense (7), confident (1) versus panicky (7), and finally relaxed (1) versus frightened (7) (Smith et al. 2012). Mean difference tests reveal between-group differences in perceived threat. Participants in the high threat condition group felt significantly more threatened than those in the no threat condition group (M_no threat_ = 2.5224, SD_no threat_ = 1.1589, M_threat_ = 5.3618, SD_threat_ = 1.3856, t = 14.628, p < 0.01). We ensured we only included cases in which participants only felt threatened in the economic crisis condition (and vice versa) by excluding cases with scores lower than 5 in the threat and higher than 3 in the no threat condition. In summary, the manipulation by the fictitious newspaper article and subsequent description of the feelings was successful.^4^ Table 3 shows exemplary quotes of our participants indicating their imagination of the threat or no-threat scenario.

**Table 3:** Exemplary quotes of the experiment’s participants when faced with the scenario

Experimental group	Firm size (years of business)	Quote
Threat	2 (8 years)	“It would be terrifying because all the hard work I did all these years, and the reputation I got to my customers will go down the drain for me with no support at all.”
Threat	2 (3 years)	“I would feel desperate and anxious due to this. I would feel sad for myself and my employees and would be looking in every way possible to get support for my business. It wouldn’t be a good feeling, and fear of the unknown would consume me every day.”
Threat	2 (6 years)	“I know exactly what it feels like. […] If my business is doing badly because of something I am doing (or not doing), that is fine because failure is completely due to my own fault. COVID-19 is a different beast. It’s too large for me to have any influence over it, and it’s the most powerful external influence I’ve encountered so far.”
Threat	10 (5 years)	“I would feel terrible, especially if my employees were relying upon me. There is no worse feeling than that of being the one who let people down, even if it’s because of something out of your control like a pandemic.”
Threat	15 (7 years)	“This will make me feel bad and I will look for a way to start a new business that will suit the current economic and health conditions.”
Control	4 (10 years)	“I would feel relieved and lucky that my business did not lose customers or revenue. Now that COVID-19 is over, I would feel inspired and motivated to continue to build my business. I would look for avenues and opportunities to expand, since I no longer run the risks caused by the pandemic.”
Control	2 (5 years)	“I feel like it would be an enormous relief. I would be proud that I was able to weather the storm of a world pandemic and would be more confident that my business could thrive in a world where we are past that challenge.”
Control	40 (5 years)	“Provided my business is not affected by the pandemic, I’ll I would greatly appreciate. I would use this as an opportunity to take the advantage I have over other businesses.”
Control	10 (5 years)	“I would feel really relieved that there are no more additional risks to my company which I have no control over. Plus, I could now look forward to expanding my business with more vigor knowing that the pandemic is behind us. This would encourage investors to invest more money and consumers to spend more money on business and services.”
Control	7 (9 years)	“I feel stronger for having gone through the pandemic. I feel my business is resilient that it managed to thrive during such a difficult time for the world. “

Firm size: number of full-time employees

### Dependent variable: effectual vs. goal-directed networking

After participants were confronted with the *threat* or *no threat* condition, they were asked which contacts in their network they would turn to in this situation (see Smith et al. 2012). Each person’s initials were listed to identify each person at a later time point (t1). The number of contacts whom the participants could list was limited to ten. This name generator process is an established and widely used measurement in network research (e.g., Burt 1982; Smith et al. 2012, 2020; Mannucci and Perry-Smith 2021). It worked as cognitive preparation to explain the rationale behind the activation of contacts.

Despite initial conceptualizations of the *goal-directed networking* of entrepreneurs being a counterpart to *effectual networking* (Engel et al. 2017), there is, to our knowledge, no study operationalizing this concept. Scanning the general networking literature, we identified and adapted the *objectification scale* of Gruenfeld et al. (2008) to the desired purpose. The instrumentality assumption (i.e., considering a single person as a “means to an end” (Gruenfeld et al. 2008, p. 111) is especially relevant for the definition of *objectification*, which is close to the definition of *goal-directed networking* (Engel et al. 2017, p. 46). In the context of network measurements, this established scale has been applied by Shea and Fitzsimons (2016), among others. Properties of *goal-directed networking,* such as selecting future contacts based on their expected future value or attempting to efficiently contact the desired partners (Engel et al. 2017), would result in higher values on the objectification scale. The description “Based on the situation described in the article, please state your agreement with the following statements.” captured items such as “I think more about what my contacts can do for me than what I can do for them,” “I get in touch with people based on how useful they are to me,” and “Networking is important to me because it helps me accomplish my goals.” These items were to be answered on a 7-point Likert scale anchored with *strongly disagree* (1) and *strongly agree* (7) with a neutral option of *neither agree nor disagree* (4). Reliability analysis results in values of *Cronbach’s alpha* above 0.7, indicating acceptable internal consistency of the scales.

### Independent variable: subjective socioeconomic status

In line with prior experiments on social inequality (Smith et al. 2012), the participants were not randomly assigned to high or low *socioeconomic status* conditions but self-assigned their perceived rank in a social hierarchy (Adler et al. 2000; Goodman et al. 2001).^5^ We ensured a high variance in socioeconomic status among the participants in the prescreening process by inviting participants on the Prolific platform from both ends of the social hierarchy. The measurement of socioeconomic status can be difficult due to the overestimation of individuals to the middle class, lack of information on household income, or reluctance to divulge this sensitive information (Smith et al. 2012). Therefore, to measure socioeconomic status, this experiment used the established measure of subjective status by Goodman et al. (2001), in which participants rated their status on a ladder. The instruction was, “At the top of the ladder are the people who are the best off, those who have the most money, most education, and best jobs. At the bottom are the people who are the worst off, those who have the least money, least education, worst jobs, or no job. Please place an ‘X’ on the rung (represented horizontally below) that best represents where you think you stand on the ladder.” Participants could select from ten rungs where the tenth represented the highest social class. This subjective assessment of socioeconomic status can be a better predictor of cognition and behavior than the measure of *objective social class* (e.g., Adler et al. 2000; Kraus et al. 2009).

Nevertheless, to test whether the subjective assessment of socioeconomic status is consistent with objective social class*,* we run correlations with the participant’s household income, highest educational attainment, and occupation (Adler et al. 2000). Our results confirm that subjective socioeconomic status and objective social class are strongly positively correlated (r = 0.590, p < 0.01). To plant the concept of socioeconomic status in participants’ minds and accordingly influence further experiment processing, this measure was deployed at the beginning of t_1_.

### Additional measures

We capture further details about the network's structure to understand our main results through additional analyses better. To measure relationships within the activated network, each participant was shown a matrix with the aforementioned initials. The participants next indicated the lack of a relationship by annotating the respective contact with an “N”, with an “A” if the contact could be described as an acquaintance, and a “C” to represent a close relationship with the contact. The procedure makes it easy to identify if the named contacts are relatively *strong* (close relation) or *weak ties* (acquaintance relation). Subsequently, the relationships between the network contacts themselves should be described according to the same principle. Again, the participants were tasked to mark the cells of a matrix with “N,” “A” or “C,” depending on the relationship between the people represented (Smith et al. 2012). Using the two matrices, we could then calculate several measures describing the activated network per participant: *network size*, *network density*, and *network constraint*.

The size of the activated network is the number of people enumerated. It is assumed that these are the people who spontaneously come to the participants’ minds following the previous manipulation (Moore 1990; Smith et al. 2012). The maximum possible number of connections between ties was calculated first to establish each participant’s network density. The number of ties depended on the number of persons mentioned and was computed as follows:

$$Potential \,Connections= \frac{n*(n-1)}{2}$$

where n is the number of named contacts of each contact. The network density was calculated by dividing the actual number of connections in the network by the above value.

$$Network\, Density= \frac{Actual \,Connections}{Potential\, Connections}$$

The *network constraint* describes the extent to which the network of a person *i* is directly or indirectly involved in the relationship of person *i* to contact *j* (Burt 2004). Network constraint thus describes the connections, resources, energy, or information a person, directly and indirectly, shares with each network contact (Smith et al. 2012). The following formula was used to calculate the *network constraint* reflecting the work of Burt (2004):

$${C}_{i}= {\sum }_{i} {\left[{p}_{ij}+ {\sum }_{q} {p}_{iq}{p}_{qj }\right]}^{2}$$

Calculating the *network constraint* theoretically results in values between 0 and 1, and greater than 1 in small ego networks (Everett and Borgatti 2020). Higher values represent more network constraints. In this sense, individuals with higher constraint values have networks where the network partners know each other directly or indirectly. Thus, high constraint values are often found in small networks with few contacts. At the same time, high constraint values can limit the identification of alternative ideas or different sources of support since few structural holes arise that promote access to unique information or other resources. Constraint is closely related to the concepts of network size and density (Burt 2004; Smith et al. 2012; Everett and Borgatti 2020). In the sample, constraint has values between 0.125 and 1.125, a mean of M = 0.7274, and a standard deviation of SD = 0.2627.

## Results

### Hypothesis testing

To verify the random assignment of participants to one of the two experimental groups, an ANOVA was performed (Gielnik et al. 2014; Hsu et al. 2019). For this purpose, manipulations were used as the dependent variable, while the demographics and control variables relating to the participants were coefficients. All *p* ratings were greater than 0.05, indicating no significant differences between the groups and successful randomization. Randomization compensates for individual differences or alternative explanations other than any resulting from the manipulation itself (Hsu et al. 2019). Consequently, neither control nor demographic variables have to be included in the ongoing procedure.

First, mean difference tests were used to examine between-group differences. The tests examine how the network activation changes due to a threat. Hypothesis 1 proposes that entrepreneurs are more goal-directed in their networking behavior in the threat condition. Goal-directedness in networking measures the degree to which contacts are a means to an end to ego (i.e., objectification) (Gruenfeld et al. 2008). Effectual networking proposes that entrepreneurs apply networking behavior based on reciprocity and serendipity rather than predefined goals (Engel et al. 2017). While economic threat situations involve high levels of uncertainty, we argue that the networking response is goal-directed (fulfilling a specific purpose). Our results show that the goal-directed networking behavior of entrepreneurs is not significantly different under threat conditions than when there is no threat (M_no threat_ = 3.0889, M_threat_ = 3.0645 p = 0.898).

**Fig. 2:**
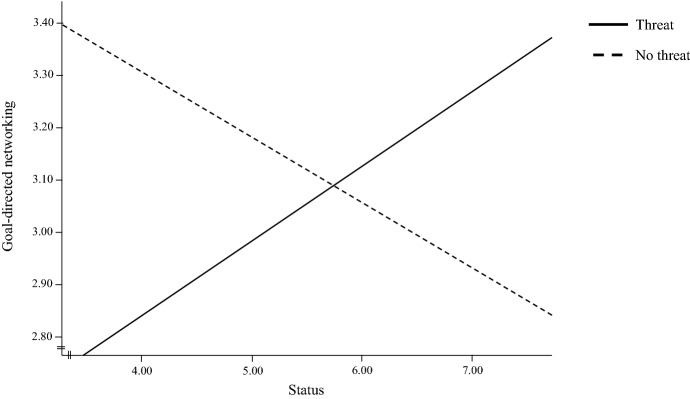
Interaction effect of threat on the relationship between socioeconomic status and goal-directed networking

**Fig. 3:**
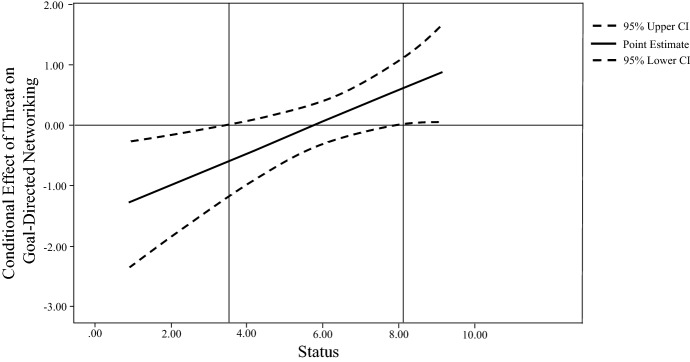
Significance areas of the conditional effect on goal-directed networking

Hypothesis 2 suggested that when differentiating between entrepreneurs on the basis of individual socioeconomic status, goal-directed networking behavior significantly differs between the threat and no threat conditions. We applied regression and moderation analysis, including a bootstrapping procedure, to assess the interaction effect (Table 4 and Fig. 2). In line with our hypothesis, we find that the socioeconomic status of entrepreneurs significantly moderates their goal-directed networking behavior under a threat condition (b = 0.2646; p < 0.0151; LLCI: 0.0520, ULCI: 0.4772). Investigation of the confidence interval areas of the interaction effect revealed the differences in goal-directed networking due to threat were especially significant for very low levels (status < 3.5355) and high levels (status > 8.2405) of socioeconomic status. The result indicates that low-status entrepreneurs significantly reduce their objectification of ties under a threat condition. In contrast, entrepreneurs of high status are significantly more likely to approach ties in a more goal-directed fashion. The analysis of the interaction’s significance areas is illustrated in Fig. 3.

**Table 4:** Regression on socioeconomic status moderating the effect of manipulated threat on goal-directed networking

	Model 1	Model 2	Model 3	Model 4 (robustness check)
Threat	– 0.024 (0.189)	– 0.023 (0.190)	– 1.515* (0.633)	– 1.494*
Socioeconomic status		0.007 (0.055)	– 0.123 (0.075)	– 0.126 (0.075)
Threat × Socioeconomic status			0.265* (0.107)	0.258* (0.109)
Gender				– 0.083 (0.179)
Age				– 0.010 (0.007)
Firm size				0.145 (0.088)
Firm age				– 0.266 (0.800)
Constant	3.089 (0.135)	3.049 (0.341)	3.795 (0.451)	4.368 (0.630)
R^2a^	0.000	0.000	0.049	0.0891

Dependent variable: Goal-directed networking; *p < 0.05, **p < 0.01; Standard errors in parenthesis; N = 122

### Robustness tests and post-hoc-analysis

Although participants were randomly assigned to the threat and no threat condition, which argues for a so-called randomized experiment, this experimental design can be classified as a hybrid design. These hybrid designs test more complicated experimental models in which several predictor variables, in this case, threat/no threat, socioeconomic status, and networking behavior, are examined. Hybrid designs are a combination of randomized and quasi-experiments. A characterization is possible because not all predictor variables are manipulated, or the participants are not randomly assigned to conditions but self-select to one of them (Hsu et al. 2017). We ensured that both treatment groups consisted of equal parts of individuals with low and high socioeconomic status. This was achieved via Prolific by sending equal parts of the survey in t_0_ to individuals of low and high socioeconomic status. Yet, to test the robustness of our results that involve non-manipulated variables, we include firm-and individual-level control variables in our regression analysis (Model 4, Table 4).

Additionally, we run bivariate correlations of all our control variables, independent variables (threat, socioeconomic status), and dependent variables of the network activation (H1–H2: Goal-Directed Networking) and network structure (network size, density, constraint), which we report together in Table 5. The correlations reveal interesting relationships that were not the focus of our study but informed the interpretation of our results. For instance, the density of strong ties in entrepreneurial networks is negatively correlated with goal-directed networking (b = – 0.225; p < 0.05).

**Table 5:** Descriptive statistics and correlations of control variables, manipulation, and all dependent variables

	Mean	S.D	1	2	3	4	5	6	7	8	9	10	11
1. Gender ^a^	1.590	0.542	1.000										
2. Age	40.870	12.858	– 0.055	1.000									
3. Firm size^b^	0.013	1.114	0.255**	– 0.056	1.000								
4. Firm age^c^	0.032	1.194	– 0.099	– 0.147	– 0.018	1.000							
5. Threat	0.510	0.502	0.012	– 0.020	0.048	0.089	1.000						
6. Socioeconomic status	5.640	1.744	0.026	0.036	0.095	0.019	– 0.044	1.000					
7. Network size	4.010	2.403	0.079	– 0.165	0.019	– 0.039	– 0.106	0.174	1.000				
8. Strong ties density	0.523	0.294	0.034	0.026	0.055	– 0.110	0.178*	– 0.029	– 0.225*	1.000			
9. Total density	0.801	0.247	0.076	– 0.066	0.088	– 0.049	0.194*	0.029	– 0.158	0.558**	1.000		
10. Network constraint	0.727	0.263	– 0.044	0.090	0.040	– 0.040	0.147	– 0.063	– 0.738**	0.291**	0.431**	1.000	
11. Goal networking	3.077	1.042	0.012	– 0.108	0.178*	– 0.007	– 0.012	0.012	0.013	– 0.225*	– 0.092	0.037	1.000

*p < 0.05, ** p < 0.01; N = 122

^a^Gender is coded as 1 = female vs. 2 = male

^b^Firm size is measured as standardized number of employees

^c^Firm age is measured as standardized measure of business duration

Furthermore, we ran several forms of post-hoc-analysis, some of which we report below. Research on the qualitative characteristics of a network addresses the difference between strong and weak ties (Granovetter 1973). While weak ties provide access to new information and serve the purpose of idea generation (Perry-Smith 2006; Perry-Smith and Mannucci 2017), strong ties provide support, trust, and constructive feedback (McFadyen and Cannella 2004; Sosa 2011; Perry-Smith and Mannucci 2017; Mannucci and Perry-Smith 2021). Entrepreneurs are more likely to activate strong ties under threat conditions (M_no threat_ = 0.4700; M_threat_: 0.5744, p < 0.05). Additionally, in response to a threat, entrepreneurs activate networks with a lower proportion of business contacts compared to other contacts such as family and friends (M_no threat_ = 0.4880, M_threat_ = 0.3254, p < 0.05).

We also test network opening by considering network density and constraint; the former measures the connections between network ties. The more interconnectedness is present in a network, the more closed the network structure is. The *overall network density* of the activated network in the *threat* scenario is significantly higher than that of the networks in the *no-threat* condition (M_no threat_ = 0.7522, M_threat_ = 0.8476, p < 0.05). The conclusion is that entrepreneurs activate more-closed networks during threat situations. *Network constraint* measures dependence between network actors (Burt 2004). The lower the network constraint, the more network actors occupy brokerage positions and span structural holes. The results show a similarly unexpected tendency toward network closure in the threat condition, while the positive mean differences are slightly insignificant (M_no threat_ = 0.6882, M_threat_ = 0.7654, p = 0.105). These results consistently suggest that entrepreneurs are more likely to activate a closed rather than open network when facing an economic threat.

## Discussion

The study investigates whom entrepreneurs call when facing an economic threat. By conducting a randomized experiment in the context of the economic shock triggered by the COVID-19 pandemic, the study finds differences in entrepreneurial networking based on entrepreneurs’ socioeconomic status. The results indicate that socialized modes of networking agency (i.e., independence vs. interdependence) drive the goal-directedness of entrepreneurial networking during a crisis. Specifically, our results show that, in times of a crisis, entrepreneurs of higher socioeconomic status are more likely to activate contacts as means to fulfill an end (i.e., goal-directed networking). In contrast, entrepreneurs with lower socioeconomic status tend to be less instrumental in networking when facing an economic threat and are more likely to offer support to their contacts (i.e., relational networking). The study’s post-hoc analysis shows that entrepreneurs preferably activate strong ties in their support network during a crisis. Entrepreneurs also tighten their network structure during crises as the density of the activated network increases in the threat condition. Taking into account the exploratory nature of this study and the need for further experiments to increase the confidence in the robustness of our findings, the study provides several opportunities for future research, including further operationalization, socialization, and contextualization of entrepreneurial networking theories and research on effective crisis responses for entrepreneurs.

### Theoretical and methodological contributions

First, the study contributes to discussions about agency in entrepreneurial networking (Engel et al. 2017; Hallen et al. 2020) by introducing the socioeconomic status of entrepreneurs as informing entrepreneurial networking behavior. Counter-intuitively, our results indicate that lower-status entrepreneurs are more likely to approach contacts to provide help than to extract benefits in a crisis. Since entrepreneurs are themselves embedded in social structure, their subjective position informs their purposeful actions. In particular, as our results and prior research on individuals’ socioeconomic status show (Snibbe and Markus 2005; Kraus et al. 2009), models of interdependent vs. independent agency rely on individual social positions. Due to their higher levels of social interdependence, individuals from lower socioeconomic positions are more likely to apply communal networking tactics. In contrast, individuals from higher socioeconomic positions use their independence to apply more instrumental networking (Kraus et al. 2012). This finding is in line with prior research on power and socioeconomic status, highlighting how social actors enforce asymmetric forms of control in social relationships (Gruenfeld et al. 2008). However, it is novel to the understanding of agency in entrepreneurial networking (Engel et al. 2017) as the interaction of entrepreneurial environments with individual social positions can alter modes of agency from independence to interdependence (Kraus et al. 2009). In other words, heterogeneity in entrepreneurial networking might stem from founders’ differences in socioeconomic backgrounds. As these backgrounds encompass individually experienced environments, this study suggests that theories of entrepreneurial networking might benefit from considering the interaction of subjective imprints from past environments and characteristics of recent environments.

Second, the study contributes to emerging research on entrepreneurial networking by testing one of the theory’s core assumptions of environmental circumstances and goal-directed networking (Engel et al. 2017) by integrating previous work on the objectification of contacts (Gruenfeld et al. 2008). Specifically, this study’s application of the objectification scale is one of the first attempts to empirically assess how and when entrepreneurs apply more or less goal-directed networking. The scale measures the degree to which individuals objectify their network contacts as means to a personal end. While the construct particularly covers the reciprocal aspect of network relationships and therefore does not cover all dimensions of entrepreneurial networking (Engel et al. 2017), it allows for testing one of the theory’s core antitheses; the goal-directedness of entrepreneurial networking. Witt (2004, p. 408) states that “entrepreneurs trying to utilize their network ties opportunistically without reciprocal offerings are bound to fail.” We argue that crises represent a boundary condition for the effectual networking logic since entrepreneurs aim for less ambiguous short-term goals such as financial stability or sales recovery. Interestingly, our results indicate that a situation of threat only changes the degree of goal-directed networking when considering entrepreneurs’ socioeconomic status. Only higher-status entrepreneurs seem to be in the position or willing to choose ties based on their usefulness. Our results echo prior research that shows networking differences during crises including the prosocial behavior of lower-status individuals (Stephens et al. 2009; Smith et al. 2012). Nevertheless, our results differ as we find that while networks are similar across entrepreneurs’ socioeconomic backgrounds, networking intentions differ. By highlighting the cognitive aspect of entrepreneurial networking, our study sheds light on the importance of the decision-making process in network activation and mobilization to better understand the mechanisms of evolving network structures (Mannucci and Perry-Smith 2021; Nai et al. 2021).

Third, the study contributes to research on entrepreneurial responses to crises. Our results emphasize that external threats influence networking decisions, particularly which sub-networks within an entrepreneurial network to activate. The challenges entrepreneurs must address change frequently, either due to dynamic environments or internal growth. The requirements of entrepreneurs in what can be extracted from their entrepreneurial networks is in a state of flux. Accordingly, for entrepreneurs, network agency involves the intentional activation of different network parts depending on specific situations (Witt 2004; Maurer and Ebers 2006; Slotte-Kock and Coviello 2010; Stam et al. 2014). We propose that selecting appropriate ties in an entrepreneurial network has important implications for entrepreneurs. An example might be activating different ties to identify and evaluate opportunities (Mannucci and Perry-Smith 2021). Prior literature on entrepreneurs’ responses to crises, and to COVID-19, in particular, reports that social support systems are critical (Doern et al. 2019; Muñoz et al. 2019; Pollack et al. 2012). Yet, there are, to our knowledge, no studies that explicitly investigate entrepreneurs’ networking responses in crises. The results of our post-hoc analysis show that entrepreneurs primarily activate dense networks involving strong ties when facing an economic threat. Combined with our findings on goal-directed networking, these results indicate that entrepreneurs’ support from (and to) social contacts during a crisis depends on their success in preemptively building dense networks, including strong ties they can later rely on (Doern et al. 2019; Williams et al. 2017).

### Limitations and future research

Our study undertakes, to the best of our knowledge, the first empirical attempt to assess the role of socioeconomic status in entrepreneurial networking. While we undertook several measures to present robust results, such as conducting a randomized preregistered experiment, the study is still subject to limitations. Particularly, our findings only partially match our expected results, and our sample of entrepreneurs on the crowdsourcing platform Prolific might lack external validity. Accordingly, we underscore the need to interpret the results of our study against the background of the study’s explorative nature. Like in other exploratory studies (e.g., Homburg et al. 2014), the novelty of the empirical attempt or the unexpected findings represent unique opportunities for future research. Accordingly, we derive several avenues for future research, partially as a response to some of the limitations and unexpected findings of the current study (see Table 6).

**Table 6:** Future research opportunities and challenges

1. Bring the diversity of entrepreneurs to the discussion: Socioeconomic status and entrepreneurial networking
How do socioeconomic status backgrounds influence the evolution of entrepreneurial network structures?
Prior research indicates that socioeconomic status is related to greater networks (e.g., Smith et al. 2012). When and how does socioeconomic status influence the formation of new network ties?
Status-heterophilic interactions: What happens if there are differences in power or status between entrepreneurial network actors (e.g., Gruenfeld et al. 2008)? Do norms of reciprocity in entrepreneurial networking hold, or are acts of deference towards the higher-status actors more likely?
What are other environmental cues that might activate status-different cognition?
Status homophily: Is the structure of entrepreneurial networks subject to status homophily (in which new tie formation is due to similar socioeconomic status)? What are the consequences of status-homophilic networks?
Conduct intersectional studies investigating the interplay of e.g., class, gender, and ethnic backgrounds on entrepreneurial networking
2. Draw attention to dynamic sub-network activation: Environmental cues and entrepreneurial networking
How do different forms of uncertainty influence entrepreneurial networking (McKelvie et al. 2011)?
Do different tasks or needs (creative vs. standardized demands) change which sub-networks entrepreneurs activate?
How do external enablers influence the activation of specific sub-networks (Davidsson et al. 2020)?
In which way does the entrepreneurial network structure change due to environmental cues?
3. Build bridges: Multiple levels of entrepreneurial networking
How do founders shape the ego-network structure of their ventures?
What is the relationship between the individual socioeconomic status of founders and the organizational status of their ventures?
What happens if there is heterogeneity in the goal-directedness of networking within an entrepreneurial team?
When do entrepreneurial teams increase biases due to socioeconomic status backgrounds (e.g., for gender biases in networks: Abraham 2020)?
What is the role of social positions for networking in different cultural and economic contexts (e.g. emerging economy, Busch and Barkema, 2022a, b)?
4. Mind actual network outcomes: The impact of support networks in crises
How do other actors in the entrepreneurial network respond when entrepreneurs seek their help? What resources do they provide, and under which terms?
Do entrepreneurs invest the necessary resources to be able to rely on support networks during crises?
What is the actual outcome of entrepreneurial networking in situations of economic threat?
5. Develop new measures: Operationalization of entrepreneurial networking
Applying and replicating the Gruenfeld et al. (2008) scale from this study to empirically seize research opportunities in entrepreneurial networking (with a focus on questions of goal-directed networking)
Developing a comprehensive scale on entrepreneurial networking. For instance, taking Vissa (2012) as starting point to develop a measure for network broadening and deepening from an effectual stance
6. Create new manipulations: Experiments in entrepreneurial networking
Add studies on entrepreneurial networking that include laboratory conditions to establish internal validity (Hsu et al. 2017; Peer et al. 2017)
Include field studies on entrepreneurial networking to ground claims of generalizability
Manipulate socioeconomic status or reemphasize consequences of social positions for a treatment group to bolster causal claims (Mullainathan and Shafir 2014; Jetten et al. 2017)
Manipulate the available network in terms of size and structure to gauge entrepreneurial networking behavior (see Perry-Smith and Mannucci 2017)

#### Socioeconomic status and entrepreneurial networking

As research on the consequences of socioeconomic status backgrounds on entrepreneurial networking is rare, we hope that the study’s implications provide a springboard for future research opportunities. Scholars might *bring the diversity of entrepreneurs and the diversity of their backgrounds* to the entrepreneurial networking discussion. New ventures are mainly driven by their founders. For instance, our results show that entrepreneurs turn to friends and family when searching for support in an economic crisis. Furthermore, individuals naturally occupy positions that involve power or status in social systems. Based on different levels of resources, privilege, or desirability, the nature of social interactions and network structures change. Due to their communal orientation affording high levels of trust, prior research indicates that individuals from lower socioeconomic backgrounds prefer smaller but more dense networks (Smith et al. 2012). Given the discussion on the importance of weak ties and structural holes for entrepreneurial networks (Burt 2004, 2019), future research might ask how socioeconomic status backgrounds influence the evolution of entrepreneurial network structures. Our study has mainly looked at the activation of a sub-network of an existing entrepreneurial network. However, what are the status-based differences in how entrepreneurs broaden their network and form new ties (e.g., Vissa 2012)? While our study has only focused on the socioeconomic status of the focal entrepreneur, future research could look at how interactions between partners change when they are of different socioeconomic status or power. Status-asymmetrical exchange relationships often imply deferential behaviors of lower-status actors. For instance, how and why does the element of reciprocity in entrepreneurial networking change in asymmetric relationships (e.g., power differences, see Gruenfeld et al. 2008)? Building on prior work on the role of gender and ethnic minority backgrounds in entrepreneurial networking (Hanson and Blake 2009; Foley and O'Connor 2013), there is a promising opportunity for future studies to apply an intersectional lens including social class. This would shed light on the interplay of categories of minority and privilege in entrepreneurial networking.

#### Environmental cues and entrepreneurial networking

Future research might look at different situations and environmental cues that change which networks entrepreneurs activate. For instance, how do different types of entrepreneurial uncertainty influence the goal-directedness of entrepreneurial networking (e.g., McKelvie et al. 2011)? Furthermore, entrepreneurs might change their modes of networking based on the type of tasks or needs. When entrepreneurs aim to creatively generate business ideas, activated sub-networks might significantly differ compared to situations of more standardized tasks (e.g., Mannucci and Perry-Smith 2021). Based on our results, future research might investigate how external factors in the entrepreneurial environment (Davidsson et al. 2020) change entrepreneurs’ network activation and networking behavior. For instance, environmental enablers such as regulatory changes might apply to specific industries increasing the activation of affected sub-sections of entrepreneurial networks. Investigating dynamics in environmental factors can increase our understanding of evolving entrepreneurial network structures.

#### Multiple levels of entrepreneurial networking

While our study derives insights from focusing on entrepreneurs as the agents of their entrepreneurial networks, much prior research has investigated new venture networks (Milanov and Fernhaber 2009; Hallen et al. 2020). Future research might bridge levels in entrepreneurial networking by explaining how founders influence the networking behavior and network structure of their new ventures. Multilevel studies are particularly interesting against the background of our investigation on socioeconomic status backgrounds. There is a body of research on organizational status that is almost entirely segregated from research on individual social status and class (e.g., Piazza and Castellucci 2014). Future research might look at the intersection of the individual socioeconomic status of founders and the organizational status of their ventures. Another opportunity for future research is the investigation of entrepreneurial teams. What happens if there is heterogeneity in the goal-directedness of networking within an entrepreneurial team? When do entrepreneurial teams increase biases due to socioeconomic status backgrounds (e.g., for gender biases in networks: Abraham 2020)? Finally, recent work on entrepreneurial networking in an emerging economy context provides further evidence that the study of entrepreneurial networking benefits from crossing levels and venturing into contexts that provide new theoretical perspectives (Busch and Barkema 2022a, b).

#### The impact of support networks in crises

There is a need for future research to assess the actual outcomes of entrepreneurial networking. While the preferences of entrepreneurs in terms of network activation certainly influence actual network evolution, it is appropriate to be cautious about the actual network consequences. Social relationships consist of interactions between at least two actors, and the mobilization of ties requires the consent of both (Smith et al. 2012). Specifically, in economic crises, preferred partners in the support networks called upon by entrepreneurs might be more reluctant to accept invitations. This notion is backed by our findings indicating that some entrepreneurs in a crisis primarily intend to interact with contacts in their network to further their own interests. Accordingly, how do other actors in the entrepreneurial network respond when entrepreneurs seek their help? What resources do they provide, and under which terms? The results of our study are subject to interpretation when it comes to the question of whether and when new ventures benefit from entrepreneurial network responses to environmental cues. Accordingly, future research can build on our results on network activation and behavior to empirically assess the outcomes of entrepreneurial networking during economic threats. Furthermore, prior research on crisis management emphasizes the critical role of preparing resilience prior to crises (Williams et al. 2017). Accordingly, future research might investigate whether entrepreneurs invest the necessary resources to be able to rely on support networks during crises.

#### Operationalization of entrepreneurial networking

Future research might apply the Gruenfeld et al. (2008) scale to empirically test existent theoretical frameworks in entrepreneurial networking (Engel et al. 2017). The theoretical roots of the objectification construct explain the instrumentality of social contacts as a result of individual power differences. Scholars can empirically investigate other factors of privilege and minority, such as gender, race, and sexual orientation in entrepreneurial networking. Future work in entrepreneurial networking would strongly benefit from the development of a comprehensive entrepreneurial networking scale that includes additional dimensions such as the formation of new ties. For instance, the scales by Vissa (2012) might be a starting point to develop a measurement of how network broadening and deepening might look from an effectual stance.

#### Experiments in entrepreneurial networking

The integration of multiple studies to counter the weaknesses of each experimental design increases the robustness of future research. For instance, a study under laboratory conditions increases internal validity (Hsu et al. 2017; Peer et al. 2017). At the same time, additional field studies foster external validity and ground claims of generalizability (e.g., see Smith et al. 2012). Our study’s sample includes entrepreneurs and small business owners. Both groups own and run businesses, but entrepreneurs differ in their high level of innovation and focus on value creation or opportunity recognition (Filion 2021). Networking might be less relevant for small business owners, especially if they do not want to create anything new or innovative and introduce it into the market. Accordingly, samples used in future research on entrepreneurial networking should either include only entrepreneurs in the narrower sense or categorize the sample into small business owners and innovative entrepreneurs to identify differences in the actors’ preferred forms of network activation and networking.

We measured the participants' subjective socioeconomic status at two points. While socioeconomic status is difficult to manipulate, future research could explore promising treatments. Some approaches to manipulating individuals’ socioeconomic status are available from extant research. One option could be to reinforce the feeling of resource scarcity when manipulating lower socioeconomic status (Mullainathan and Shafir 2014). Another possibility would be to emphasize the participants’ position in society (Jetten et al. 2017). As methods such as Burt’s name generator might elevate a tendency to report the activation of strong ties (Marin 2004), further experimentation involving alternative measures is necessary. The issue is particularly relevant for possible research on the relationship between the activated network and networking behavior. It follows that by manipulating the activated network in terms of size and structure, future researchers could also show what information is available due to a person’s position in the network and investigate the roots of that position. Mannucci and Perry-Smith (2021) offer an exciting approach to this issue.

## Conclusion

On a more general note, this study highlights the different modes of networking agency of entrepreneurs in a crisis response scenario. While activating social support might be only one dimension in entrepreneurs’ complex responses to crises such as COVID-19, we provide evidence that entrepreneurs decide carefully whom to call in times of crisis. The network response of entrepreneurs in a crisis might be another piece of the puzzle of why and under which circumstances entrepreneurs can turn existential economic threats into entrepreneurial opportunities. Yet, we also shed light on the circumstance that crises often reproduce social inequality. Entrepreneurs in disadvantaged positions face additional strains in overcoming economic crises. Accordingly, future research might put additional emphasis on understanding inequal access to entrepreneurial opportunities during crises. For instance, future research might investigate how entrepreneurs from lower socioeconomic backgrounds overcome these additional challenges during crises. At the same time, our results indicate that entrepreneurs from lower socioeconomic status backgrounds support others during crises. As such, the focus on maintaining relationships during crises might be a unique strength that results in ongoing social and economic value beyond the crisis.

## Data Availability

The study’s experiment is preregistered. As part of our submission, the editor received access to the authors’ pre-experiment statements on AsPredicted.org in order to ensure procedural transparency. The data of this study are available from the corresponding author, Andreas Kuckertz, upon reasonable request.

## Footnotes

1 Both quotes are responses from our study participants to how their business has been affected during the pandemic.

2 While most scholars agree that social class encompasses objective (income, education, occupation) and subjective dimensions (Côté 2011; Loignon and Woehr 2018; Kish-Gephart et al. 2022), the definition of socioeconomic status is more controversial. In this study, we follow Kraus et al. (2009) and understand subjective socioeconomic status as one (of many) components of social class that captures individuals ‘ subjective rank vis à vis others in society.

3 In addition, we recorded how plausible the respondents considered the research procedure and how accurately it represented real-life situations experienced personally. The results confirm the research design, which was then adopted for the experiment. We ensured that the entrepreneurs involved in the pre-test were not in the later sample. We can provide data on the pre-tests upon request.

4 To check whether individual participants accurately read and understood the text manipulation, they were asked "Were you told in the news article that the COVID-19 crisis is over?" after measuring how threatened they felt. They could answer with "yes" and "no". Another 15 persons failed this manipulation check and were therefore removed from the sample.

5 Hybrid experimental designs are a combination of randomized and quasi-experiments in which not all predictor variables are manipulated, or the participants are not randomly assigned to conditions, but self-select to one of them (Hsu et al. 2017). Since an individual’s status is hard to manipulate in an experimental design, we follow prior research in adding a self-reported predictor variable (Smith et al. 2012; Mittal and Griskevicius 2014).
